# Menthae Herba Attenuates Neuroinflammation by Regulating CREB/Nrf2/HO-1 Pathway in BV2 Microglial Cells

**DOI:** 10.3390/antiox11040649

**Published:** 2022-03-28

**Authors:** Yeo Jin Park, Hye Jin Yang, Wei Li, You-Chang Oh, Younghoon Go

**Affiliations:** 1 Korean Medicine Life Science, University of Science and Technology, Daejeon 34054, Korea; pyjin5526@kiom.re.kr; 2 Korean Medicine (KM)-Application Center, Korea Institute of Oriental Medicine (KIOM), Daegu 41062, Korea; hjyang@kiom.re.kr (H.J.Y.); liwei1986@kiom.re.kr (W.L.); ulivuli@kiom.re.kr (Y.-C.O.)

**Keywords:** BV2, anti-neuroinflammation, ROS, NF-κB, HO-1

## Abstract

Chronic inflammation and oxidative stress cause microglia to be abnormally activated in the brain, resulting in neurodegenerative diseases such as Alzheimer’s disease (AD). Menthae Herba (MH) has been widely used as a medicinal plant with antimicrobial, anti-inflammatory, and antioxidant properties. In this study, we sought to evaluate the effects of MH on the inflammatory response and possible molecular mechanisms in microglia stimulated with lipopolysaccharide (LPS). Transcriptional and translational expression levels of the proinflammatory factors were measured using ELISA, RT-qPCR, and Western blot analysis. MH extract inhibited the production of proinflammatory enzymes and mediators nitric oxide (NO), NO synthase, cyclooxygenase-2, tumor necrosis factor-α, and interleukin-6 in LPS-stimulated cells. Our molecular mechanism study showed that MH inhibited the production of reactive oxygen species (ROS) and the phosphorylation of mitogen-activated protein kinase and nuclear factor (NF)-κB. In contrast, MH activated HO-1 and its transcriptional factors, cAMP response element-binding protein (CREB), and the nuclear factor erythroid 2-related factor 2 (Nrf2) signaling pathways. Thus, MH reduces ROS and NF-κB-mediated inflammatory signaling and induces CREB/Nrf2/HO-1-related antioxidant signaling in microglia. Together, these results may provide specific prospects for the therapeutic use of MH in the context of neuroinflammatory diseases, including AD.

## 1. Introduction

Along with an increase in lifespan, interest in neurodegenerative diseases and neurological health is growing, leading to a better quality of life. Accumulating evidence indicates that chronic neurological diseases, including Alzheimer’s disease (AD) and Parkinson’s disease, are closely associated with neuroinflammation [1]. Since proinflammatory responses induced by pathological factors, including reactive oxygen species (ROS) and the production and secretion of cytokines, may be harmful to normal neurons, leading to synaptic dysfunction, neuronal death, and loss of synapses [2], it is important to control the inflammatory response to prevent the progression of chronic neurodegenerative disease.

Although microglia are a primary constituent of the dedicated immune system in the brain, it has been reported that the abnormal activation of microglia is involved in multiple pathological signaling pathways [3]. As glial cells provide multiple physiological functions under basal or disease conditions, inflammatory stimuli, such as lipopolysaccharide (LPS), can induce microglia to aggravate inflammation by over-releasing proinflammatory factors, such as interleukin (IL)-1β, IL-6, tumor necrosis factor-α (TNF-α), nitric oxide (NO), and ROS, eventually causing neuronal dysfunction and cell death [4].

Increasing evidence shows that ROS are secondary messengers in microglia that develop progressive inflammation processes and may contribute to dysregulation of the immune response [5]. In pathological situations and exposure to stressful environmental stimuli, ROS production is excessively increased [6], leading to persistent and inappropriate inflammation. However, the brain defends itself against oxidative stress owing to elevated ROS generation, modest antioxidant defenses, and insufficient regeneration ability [7]. Antioxidants are both endogenous and exogenous molecules that limit the detrimental effects of free radicals in neuronal cells through detoxification [8]. Heme oxygenase (HO-1) is an antioxidant enzyme that plays a key role in protecting against oxidative damage and inflammation [9]. HO-1 ultimately induces bilirubin from heme, and its product is a powerful antioxidant [10]. Therefore, the induction of HO-1 is generally considered a defense response against neuronal damage in various inflammatory neurological conditions owing to its enzymatic function [11].

We aimed to identify anti-inflammatory drugs from natural products owing to their fewer side effects on the human body. We screened over 300 natural extracts to identify the most effective product with anti-inflammatory effects and, finally, selected one for being effective on inflammation in BV2 cells (data not shown). Menthae Herba (MH), a perennial herbaceous plant of the Lamiaceae family, is widely used in food, cosmetics, and medicines [12]. Over several millennia, MH has been used traditionally in Korea and China to treat various diseases, including fever, headache, sore throat, thrush, and rubella [13]. In addition, pharmacological studies have shown that MH possesses various biological activities, including antimicrobial, anti-inflammatory, antioxidant, antitumor, gastrointestinal protective, and hepatoprotective activities [14]. MH consists of various volatile compounds that mainly contain monoterpenoids, menthone, and menthol, which may contribute to its therapeutic activities [15,16]. Although several pharmacological studies of MH have revealed its anti-inflammatory activities, its effects on neuroinflammation have not previously been reported in detail. In this study, we evaluated the anti-inflammatory and antioxidant effects of an ethanolic extract of MH in LPS-stimulated BV2 microglial cells.

## 2. Materials and Methods

### 2.1. Materials and Reagents

Dulbecco’s modified Eagle’s medium (DMEM) was obtained from Welgne Inc. (Gyeongsan, Korea). Fetal bovine serum and antibiotics were purchased from Hyclone (Logan, UT, USA). LPS, dexamethasone (DEX), and dimethyl sulfoxide (DMSO) were purchased from Sigma-Aldrich Co. (St. Louis, MO, USA). Bovine serum albumin (BSA) was purchased from GenDEPOT (Katy, TX, USA). Cell counting kits (CCK) were obtained from Dojindo Molecular Technologies, Inc. (Kumamoto, Japan). A RNeasy Mini Kit for RNA extraction was obtained from Qiagen (Hilden, Germany). A Maxima First Strand cDNA Synthesis Kit for RT-qPCR and PowerTrack SYBR Green Master Mix were obtained from Thermo Scientific (Waltham, MA, USA). Oligonucleotide primers for real-time quantitative PCR were synthesized by Cosmogenetech (Seoul, Korea). Enzyme-linked immunosorbent assay (ELISA) kits were obtained from R&D Systems (Minneapolis, MN, USA). Primary antibodies were purchased from Cell Signaling Technology, Inc. (Boston, MA, USA). Horseradish peroxidase (HRP)-conjugated secondary antibodies were purchased from Bethyl Laboratories Inc. (Montgomery, TX, USA). Cell culture dishes and plates were purchased from Sarstedt (Nümbrecht, Germany). MH extract (freeze-dried powder) was provided from the Korea Plant Extract Bank (Ochang, Korea), which was extracted in 95% ethanol by reflux and dissolved in DMSO and stored at −20 °C for bioassay use. The standard compound (±)menthol used for analysis was purchased from Sigma-Aldrich Co. (St. Louis, MO, USA), and the purity was above 98%. High-Performance Liquid Chromatography (HPLC)-grade acetonitrile and methanol were obtained from Merck KGaA (Darmstadt, Germany). ACS reagent-grade formic acid was purchased from Sigma-Aldrich Co. (St. Louis, MO, USA). The ultrapure water used for HPLC analysis was purified by UP Quality 18.2 MΩ cm^−1^ using a Puris-Evo UP Water System equipped with Evo-UP Dio VFT and Evo-ROP Dico20 (Mirae ST Co., Ltd., Anyang, Gyeonggi-do, Korea).

### 2.2. High-Performance Liquid Chromatography-Charged Aerosol Detector (HPLC-CAD) Analysis

A stock solution of menthol was prepared at a concentration of 1000 μg/mL in methanol. Then, it was diluted to a final concentration of 500 μg/mL to prepare a working solution. The analytical sample (MH extract) was prepared in methanol at a concentration of 10 mg/mL. Then, menthol and the sample solution were filtered through a 0.22 μm PTFE membrane filter (Whatman International Ltd., Maidstone, UK) prior to injection into the HPLC-CAD system. A chromatographic analysis was performed using a charged aerosol detector (CAD) coupled with HPLC to identify menthol, the main component of MH. The HPLC system Thermo Dionex UltiMate 3000 was equipped with a binary pump, an autosampler, a column oven, a diode array UV/VIS detector, and a CAD (Thermo Fisher Scientific, San Jose, CA, USA). The analysis was conducted according to a previously reported method [17]. The separation was carried out using a Gemini C18 column (4.6 × 150 mm, 5 µm, Phenomenex, Torrance, CA, USA). The temperature of the column oven was maintained at 35 °C, and the injection volume of each sample was 10 µL. The mobile phase consisted of 0.1% formic acid (*v*/*v*) in water (A) and acetonitrile (B), and to improve chromatographic separation capacity, the gradient elution system was programmed at a flow rate of 0.8 mL/min as follows: 80% B, 0–5 min; 80–100% B, 5–15 min; 100% B, 15–25 min; 100–80% B, 25–26 min; and 80% B, 26–35 min. The CAD channel was optimized using the following parameters: power function, 1.00; data collection rate, 10 Hz; filter, 0.1; peak width, 0.02 min; control evaporator temperature, low; and wait ready, ±5.0 K. All data acquisition and analyses were performed using Dionex Chromelon software.

### 2.3. Cell Culture and Drug Treatment

BV2 microglial cells were provided by Professor Kyoungho Suk of Kyungpook National University (Daegu, Korea). The cells were cultured in DMEM containing 1% antibiotics and 10% FBS in a humidified atmosphere of 5% CO_2_ at 37 °C. The cells were pretreated with MH extract (10, 25, or 50 μg/mL) or DEX (10 μM) for 1 h and then stimulated with 100 ng/mL LPS for the indicated periods.

### 2.4. Cell Viability Test

BV2 microglial cells were plated in 96-well plates at a density of $5\times 10^{4}$ cells/well for 18 h. To determine the cytotoxic effect of the drug, the cells were incubated with the indicated concentrations of the MH extract for 24 h. After treatment with 10 μL of CCK solution per well for 1 h, the cell viability was analyzed using an ELISA reader at 450 nm.

### 2.5. Determination of NO Production

NO production in the culture supernatants of the BV2 cells was evaluated by measuring the absorbance of nitrite at the 550 nm wavelength. Cells ($5\times 10^{4}$ cells/well) were seeded in 96-well plates, pretreated with the indicated concentrations of MH extract for 1 h, and then stimulated with LPS for 24 h. Conditioned media were collected, added to the same volume of Griess reagent, and measured spectrophotometrically. Sodium nitrite was used as the standard to calculate the concentration of nitrite.

### 2.6. ELISA for the Analysis of Inflammatory Cytokine Secretion

To evaluate the effects of the MH extract on the secretion of inflammatory cytokines in the culture supernatants of BV2 cells, ELISA assays for TNF-α and IL-6 were performed. The cells ($2.5\times 10^{5}$ cells/well) were plated in 24-well plates, processed, and treated with MH and LPS under the same conditions as those used in the other experiments. After 6 h of stimulation, the culture media were retrieved according to the manufacturer’s standard protocols for the ELISA assay. The amount of each inflammatory cytokine was calculated using absorbance at a wavelength of 450 nm.

### 2.7. Western Blotting for Protein Expression Measurement

For Western blot analysis, BV2 cells were plated on a six-well plate at a density of $1.5\times 10^{5}$ cells/well and cultured for 24 h. After treatment with MH extract and LPS, cultured cells were collected at predetermined times. The cells were lysed in radioimmunoprecipitation assay lysis buffer (Millipore, Burlington, MA, USA) and incubated on ice for 30 min. After centrifugation at 15,000× *g* rpm for 10 min at 4 °C, the total proteins were collected and normalized using the BCA protein assay kit (#23225, Thermo Fisher Scientific). Protein samples were separated using 10% sodium dodecyl sulfate–polyacrylamide gel electrophoresis and transferred to polyvinylidene fluoride membranes (Millipore, Burlington, MA, USA). Following blocking with 3% bovine serum albumin for 1 h at room temperature, the samples were incubated with specific primary antibodies at 4 °C. The membranes were then rinsed with Tris-buffered saline (TBS)-T containing 0.1% Tween 20 and incubated with horseradish peroxidase-conjugated secondary antibodies. Protein bands were detected using Alliance Q9 mini (UVITEC, Cambridge, UK) and quantified using ImageJ software. 

### 2.8. RNA Isolation and RT-qPCR

Total cellular RNA was isolated using the QIAzol Lysis Reagent (Qiagen, Hilden, Germany). The purified RNA was reverse-transcribed into cDNA using the Maxima First Strand cDNA Synthesis Kit for RT-qPCR (Thermo Fisher Scientific, Waltham, MA, USA). The amplification reactions for qPCR were performed in 10-µL reaction volumes containing the primers and Power SYBR^®^ Green Master mix (Thermo Fisher Scientific) and detected using a C1000 Touch^TM^ Thermal Cycler (Bio-Rad, Hercules, CA, USA). The samples were compared using the relative CT methods. The expression of 36B4 was used for normalization and quantified using the 2^−ΔΔCq^ method. The primer sequences used are listed in Table 1.

### 2.9. Immunofluorescence

Coverslips were coated with poly-D-lysine (Gibco, CA, USA) in 24-well plates for 15 min, and then, BV2 cells were seeded onto glass coverslips for 24 h. Cells were pretreated with MH or DEX in serum-free medium for 1 h, stimulated with LPS (100 ng/mL) for 1 h, washed with phosphate-buffered saline (PBS), and fixed in cold 4% paraformaldehyde in distilled water for 30 min. Then, the cells were rinsed, permeabilized with 0.5% Triton X-100 in PBS, incubated in blocking solution for 30 min, and then probed with an NF-κB p65 antibody (Cell Signaling Technology, Inc., Danvers, MA, USA) at 4 °C for 24 h. A secondary antibody, anti-rabbit IgG (Bethyl Laboratories, Montgomery, TX, USA) labeled with Alexa 488 (1:1000) was used for the visualization of fluorescence. To stain the nuclei, the cells were treated with Hoechst 33258 (Thermo Fisher Scientific) in PBS (1:10,000) for 5 min at room temperature in the dark. After washing and mounting, stained cells were visualized using a fluorescence microscope (Lionheart FX automated microscope, BioTek, Winooski, VT, USA).

### 2.10. ROS Measurement

BV2 cells were cultured in 96-well plates ($5\times 10^{4}$ cells/well). The cells were pretreated with the indicated concentration of MH for 1 h and then stimulated with 100 ng/mL LPS for 24 h. Intracellular ROS levels were detected using ROS-sensitive probe 2′,7′-dichlorodihydrofluorescein diacetate (DCFDA, Invitrogen, D-399). DCFDA was dissolved in DMSO and further diluted before use. BV2 cells were incubated with 5-µM DCFDA staining solution in PBS in the dark for 1 h at 37 °C, then washed and imaged with a fluorescence microscope (Nikon ECLIPSE Ti-U, Nikon Co., Tokyo, Japan). Signal quantification was determined by measuring the fluorescence using a GloMax^®^ Explorer Multimode Microplate Reader (Promega). The results were presented as the relative ratio compared with untreated control cells.

### 2.11. Statistical Analysis

Data were presented as the mean ± SEM obtained from three individual experiments, and the experiments were performed at least in triplicate (*n* = 3). All data were analyzed by one-way analysis of variance (ANOVA) followed by Tukey’s honest significant difference test using GraphPad Prism 5.0 (GraphPad Software, San Diego, CA, USA). Differences were considered statistical significant at *p*-values < 0.05.

## 3. Results

### 3.1. HPLC-CAD Analysis of MH

**Figure 1:**
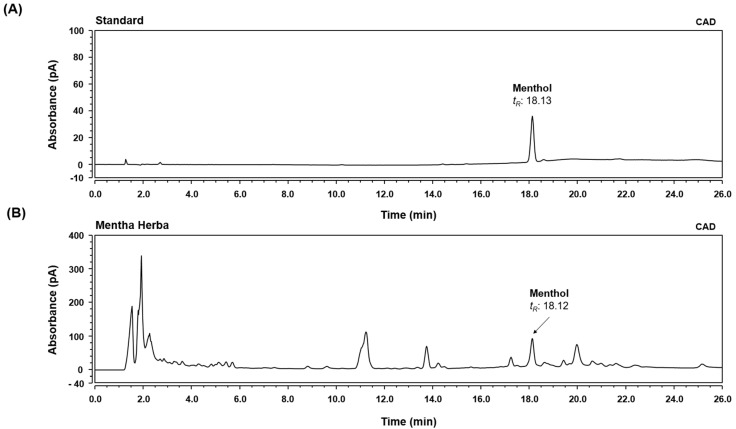
HPLC-CAD analysis of menthol in Mentha Herba (MH). CAD chromatogram of standard solution (**A**) and MH (**B**).

Menthol, the main component in MH, was determined by comparing the retention time (tR), UV spectra, and chromatogram patterns with those of the standard using a HPLC system. In addition, the detection sensitivity of the menthol component was improved using a detector to measure the amount of chemicals in the sample by generating charged aerosol particles. As shown in Figure 1, menthol was well-separated within 26 min and showed good resolution while minimizing the interference by other analytes. The retention time of the standard compound was analyzed at 18.13 min in the chromatogram. Under the same conditions, the retention times of the observed analytes in MH were 18.12 min.

### 3.2. Effects of MH on Cell Viability of BV2 Cells

**Figure 2:**
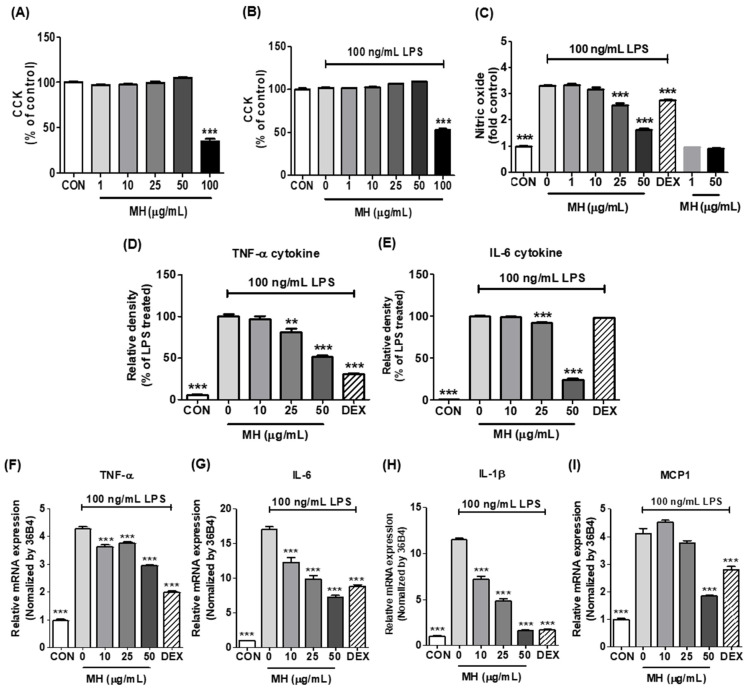
Effects of MH on the production of NO and inflammatory cytokines in LPS-stimulated BV2 cells. (**A**) Cell viability in BV2 cells treated with MH at the indicated concentrations for 24 h. (**B**) BV2 cells were treated with MH at the indicated concentrations for 1 h and then stimulated with LPS for 24 h. Cell viability was measured by the CCK assay. (**C**) BV2 cells were treated with MH at the indicated concentrations or 10 μM DEX for 1 h before stimulation with LPS for 24 h. NO production was measured by Griess reagent assays. (**D**,**E**) Inflammatory cytokines secretion and (**F**–**I**) mRNA expression of inflammatory cytokines in BV2 cells treated with MH at the indicated concentrations for 1 h and then stimulated with LPS for 6 h. Data are expressed as the mean ± SEM. ** *p* < 0.01 and *** *p* < 0.001 vs. LPS control.

As a preliminary assessment, an appropriate MH concentration was established to attenuate LPS-stimulated neuroinflammation in the absence of overt toxicity. CCK and Griess reagent assays were conducted on BV2 microglial cells in the presence or absence of LPS and the extract. As shown in the CCK assay for the cell viability test, MH was not toxic to the cells at concentrations up to 50 μg/mL when activated separately or together with LPS (Figure 2A,B). Therefore, MH was used at concentrations below 50 μg/mL in subsequent studies to eliminate the possibility of toxic effects of MH on cells.

### 3.3. MH Reduces NO Production in LPS-Stimulated BV2 Cells

BV2 cells were administered LPS (100 ng/mL), which is extensively used in inflammation studies. The Griess reagent assay showed that BV2 cells produced high concentrations of NO (a mediator of neurotoxic reactions) in the presence of LPS. When comparing the effect of MH between drug-treated groups and the LPS-treated control, NO release increased with LPS stimulation and was significantly decreased at concentrations ≥25 μg/mL MH in a dose-dependent manner. DEX, a representative anti-inflammatory drug, also slightly decreased the NO release of LPS-induced BV2 cells (Figure 2C). 

### 3.4. MH Decreases Inflammatory Cytokines and Chemokine in LPS-Stimulated BV2 Cells

To evaluate the efficacy of MH extract treatment in mitigating the proinflammatory processes triggered by LPS in microglia, we measured the production of proinflammatory cytokines, including TNF-α and IL-6. ELISA showed that LPS markedly produced and secreted TNF-α and IL-6 cytokines, while pretreatment with MH reduced their secretion in a dose-dependent manner. DEX significantly decreased the secretion of TNF-α and failed to induce IL-6 secretion (Figure 2D,E).

To evaluate whether MH extract also altered the transcriptional changes in the mRNA expression of the cytokines, we analyzed the relative gene expression of the inflammatory cytokines in BV2 cells by RT-qPCR. LPS stimulation resulted in higher mRNA expression levels of TNF-α and IL-6 in BV2 cells than those in the nontreated controls. At all concentrations tested (10, 25, and 50 μg/mL), pretreatment with the MH extract significantly decreased the expression of cytokines (Figure 2F,G). Furthermore, we examined additional proinflammatory cytokines (IL-1β and MCP-1) in BV2 cells, where LPS showed a statistically significant increase in both cytokine transcripts compared to those in the untreated control (Figure 2H,I). However, treatment with MH rescued cytokine gene expression in the cells. Specifically, it reduced IL-1β mRNA expression in a dose-dependent manner, reaching levels at 50 μg/mL comparable to those observed in the control (Figure 2H). The increased mRNA expression of MCP-1 triggered by LPS was also significantly reduced at a concentration of 50 μg/mL (Figure 2I).

### 3.5. MH Represses LPS-Mediated Expression of iNOS and COX-2

**Figure 3:**
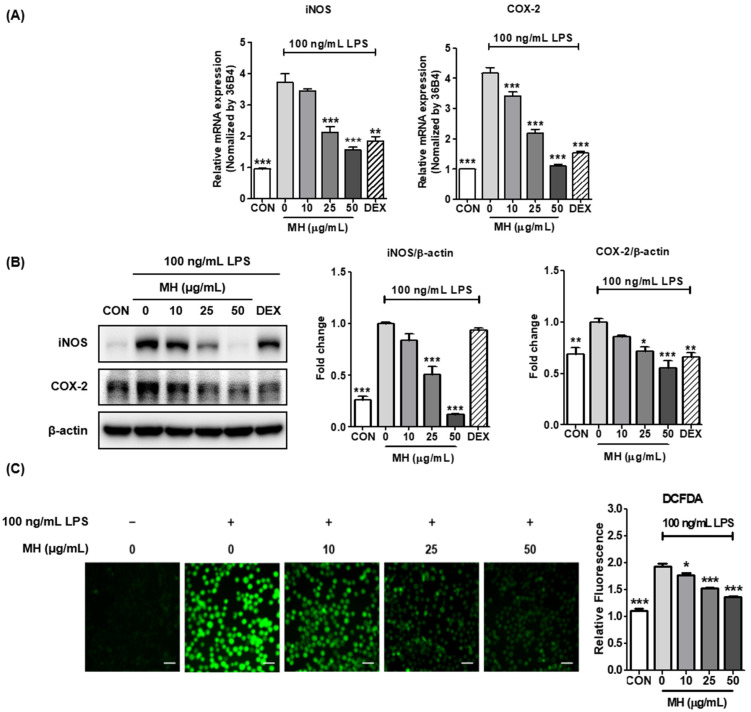
Effects of MH on (**A**) mRNA expressions and (**B**) protein levels of iNOS, COX-2, and (**C**) ROS production. The cells were preincubated with MH at the indicated concentrations or 10 μM DEX for 1 h before stimulation with LPS. The cells were stimulated with LPS for 12 h (mRNA and protein) or 18 h (ROS). The ROS levels were measured by DCFDA; the relative amount of cellular ROS was represented as a fold change over BV2 cells. Data are expressed as the mean ± SEM. * *p* < 0.05, ** *p* < 0.01, and *** *p* < 0.001 vs. LPS control. Scale bar: 100 μm.

Considering the effects of MH treatment on the attenuation of NO induction in LPS-exposed BV2 cells, we investigated whether such profitable effects were also related to transcriptional changes in the mRNA expression of inducible NOS, an enzyme that synthesizes NO. In contrast, COX-2 is an enzyme that catalyzes the production of prostaglandins (PG), resulting in the exacerbation of neuroinflammatory processes [18]. As shown in Figure 3A,B, the mRNA and protein expression levels of iNOS and COX-2 were dramatically increased in BV2 cells stimulated with LPS, demonstrating that these genes have emerged as major players in brain inflammation, and increased iNOS and COX-2 expression was associated with neurodegeneration. Furthermore, inhibitory effects on both the mRNA and protein expression of iNOS and COX-2 were observed at the indicated concentrations of MH, resulting in levels at 50 μg/mL not significantly different from the control. DEX exposure also attenuated the mRNA and protein expression of LPS-mediated iNOS and COX-2.

### 3.6. MH Inhibits ROS Production in LPS-Treated BV2 Cells

Based on the data showing that MH treatment decreased the NO, cytokine, and enzyme levels related to neuroinflammatory processes, we further tested whether MH regulates ROS production in BV2 cells. As expected, MH treatment notably decreased ROS production, which was significantly increased compared to that in the control after LPS treatment (Figure 3C). These data indicate that MH significantly downregulated the neuroinflammatory response in LPS-induced BV2 cells.

### 3.7. MH Suppresses the Transcriptional Activity of NF-κB

**Figure 4:**
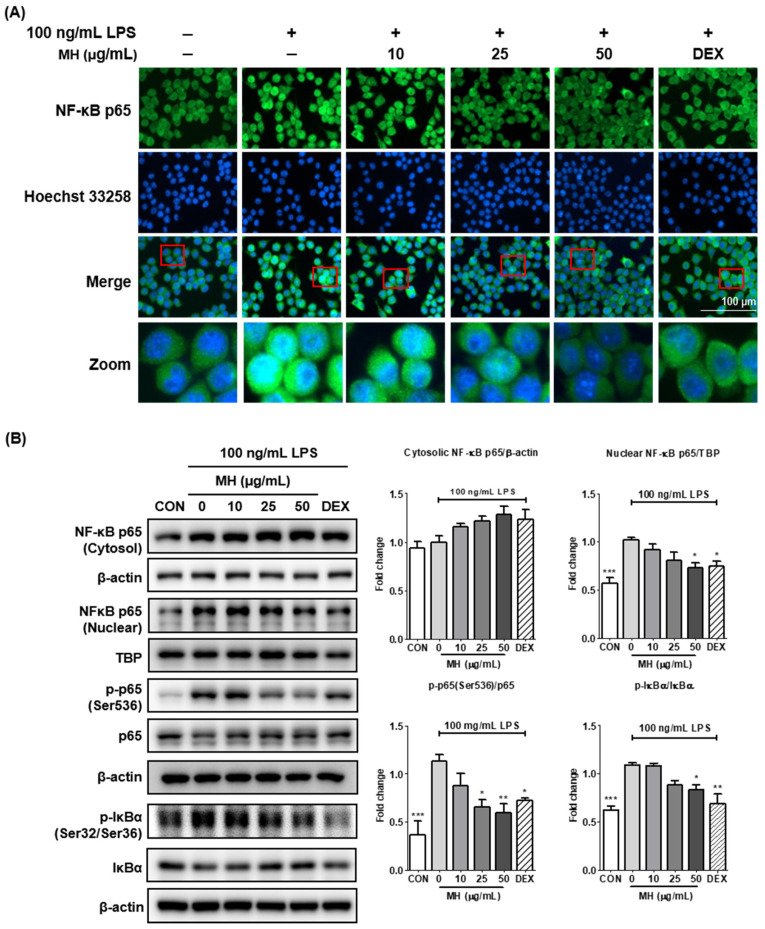
Effects of MH on the (**A**) translocation, (**B**) cytosol, nuclear protein expression, phosphorylation of NF-κB p65, and phosphorylation and degradation of IκBα. The cells were preincubated with the indicated concentration of MH or 10 μM DEX for 1 h before stimulation with LPS. The cells were stimulated with LPS for 1 h (NF-κB p65) or 30 min (IκBα). Immunofluorescence staining was used for detecting NF-κB p65 translocation. The cells were counterstained with Hoechst 33258. (**B**) β-actin was used as the cytosolic endogenous control. TBP was used as the nuclear endogenous control. Data are expressed as the mean ± SEM. * *p* < 0.05, ** *p* < 0.01, and *** *p* < 0.001 vs. LPS control.

Since the activation of nuclear factor (NF)-κB during the inflammatory response promotes the transcription of proinflammatory mediators and cytokines, we further evaluated whether MH inhibits LPS-induced NF-κB activation. To determine this, immunofluorescence staining was performed on BV2 cells treated with MH with or without stimulation with LPS for 1 h. As shown in Figure 4A, LPS stimulation increased the fluorescence intensity of NF-κB p65 in the nuclei, which was reversed in a dose-dependent manner in the presence of MH, indicating that MH inhibited the nuclear translocation of NF-κB p65. Consistent with the immunofluorescence images, the expression of NF-κB p65 in the nuclei of LPS-treated cells was markedly increased. Furthermore, the phosphorylation and degradation of IκBα were elevated by LPS treatment, indicating that NF-κB p65 was activated. However, pretreatment with MH reduced the nuclear translocation of NF-κB p65 and phosphorylation and degradation of IκBα in LPS-treated cells (Figure 4B). These findings suggest that MH may prevent neuroinflammation through inactivation of the NF-κB signaling pathway.

### 3.8. MH Inactivates MAPK Signaling

**Figure 5:**
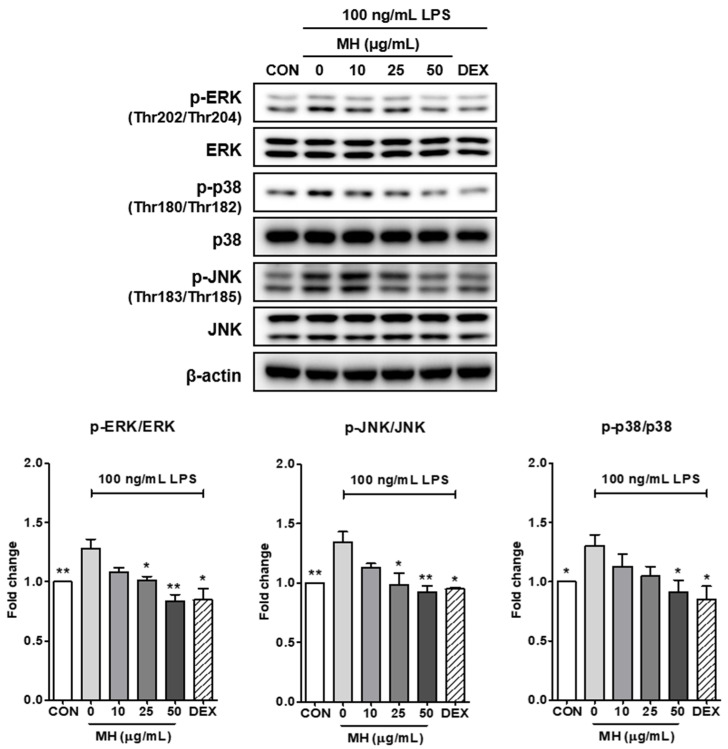
Effects of MH on the phosphorylation of ERK, p38, and JNK MAPK. Representative Western blots and densitometric quantification of p-ERK, ERK, p-p38, p38, p-JNK, and JNK expression in BV2 cells treated with LPS (100 ng/mL) alone or in combination with 3 doses of MH or left untreated (CON). The cells were preincubated with the indicated concentration of MH or 10 μM DEX for 1 h before stimulation with LPS. The cells were stimulated with LPS for 30 min. β-actin was used as an endogenous control. Data are expressed as the mean ± SEM. * *p* < 0.05 and ** *p* < 0.01 vs. LPS control.

It has been reported that NF-κB is activated by mitogen-activated protein kinases (MAPKs), including ERK, p38, and JNK [19,20]. To assess the inhibitory effect of MH on the causative molecular mechanisms of NF-κB activation in LPS-induced inflammatory conditions, we tested whether MH affected the MAPK signaling pathways. As shown in Figure 5, LPS stimulation significantly phosphorylates the main MAPK extracellular signal-regulated kinase (ERK), c-Jun NH_2_-terminal kinase (JNK), and p38, and pretreatment with MH represses the phosphorylation of MAPK in a dose-dependent manner without altering the total form. The LPS-treated cells also responded significantly to DEX exposure.

### 3.9. MH Increases HO-1 Expression through Nrf2/CREB Signaling

**Figure 6:**
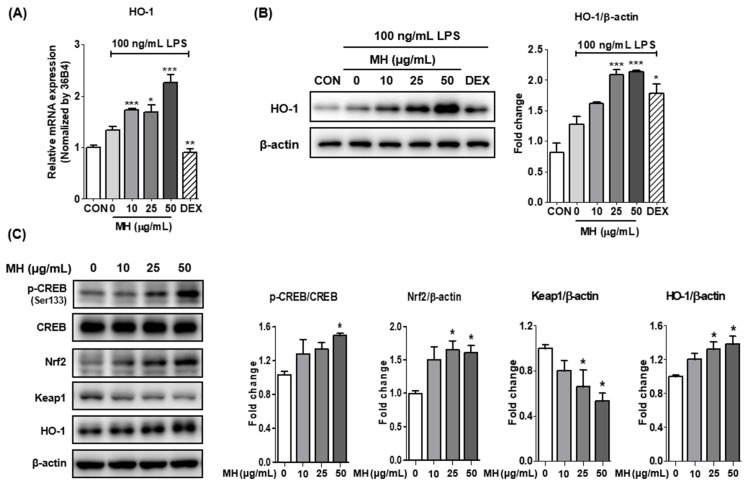
Effects of MH on antioxidative genes. (**A**) mRNA expression levels of HO-1 in BV2 cells treated with LPS and MH. (**B**) Representative Western blots and densitometric quantification of the HO-1 protein levels in BV2 cells treated with LPS and MH. The cells were preincubated with the indicated concentration of MH or 10 μM DEX for 1 h before stimulation with LPS. (**C**) Representative Western blots and densitometric quantification of CREB, phosphorylated CREB, Nrf2, and Keap1 in BV2 cells only treated with MH. β-actin was used as an endogenous control. Data are expressed as the mean ± SEM. * *p* < 0.05, ** *p* < 0.01, and *** *p* < 0.001 vs. LPS control.

To evaluate the other molecular mechanisms responsible for the anti-inflammatory effects of MH, we tested whether MH mediates the antioxidant signaling pathway. Transcriptional and translational upregulation of HO-1 was observed in BV2 cells treated with MH following stimulation with LPS (Figure 6A,B). To investigate the upstream mechanism of HO-1, we assessed the activation of the transcriptional factors of HO-1 by Western blot. According to the results of the Western blot analysis, phosphorylation of the cAMP response element-binding protein (CREB) was increased by MH in a dose-dependent manner. In addition, the degradation of Kelch-like ECH-associated protein (Keap)1 was increased by MH, indicating that nuclear factor erythroid 2-related factor 2 (Nrf2) was translocated from the cytoplasm to the nucleus (Figure 6C). These results suggest that the anti-inflammatory effects of MH are mediated by the antioxidant effect of HO-1 through the activation of CREB and Nrf2.

## 4. Discussion

It is widely accepted that inflammation is involved in numerous diseases of the whole body, especially in the brain, and contributes to neurodegenerative diseases, including AD. Under inflammatory stimuli, microglia are progressively and abnormally activated and exacerbate inflammation worsened by the overexpression of proinflammatory molecules such as IL-1β, IL-6, TNF-α, NO, and ROS, eventually resulting in neuronal dysfunction and cell death [4]. Thus, a drug that reduces neuroinflammation via microglial activation is a promising therapeutic candidate for treating neurodegenerative diseases.

Previous studies have shown the anti-inflammatory effects of *Mentha* species in activated RAW264.7 cells [21] and an asthma mouse model [22]. However, it is still unclear whether MH inhibits neuronal inflammation in the central nervous system. We demonstrated the ability of MH to inhibit LPS-stimulated microglial activation, which contributes to neurodegenerative processes. In this study, the underlying mechanisms included, but were not limited to, the downregulation of proinflammatory genes associated with the NF-κB and HO-related antioxidant signaling pathways.

Microglial cells are abnormally activated by inflammatory stimulation, such as LPS, and secrete inflammatory factors, such as NO and PG and the proinflammatory cytokines IL-6, IL-1β, and TNF-α [18]. Under oxidative stress conditions, NO participates in oxidative-reductive processes to form reactive nitrogen species, which are noxious compounds for cognitive function in the CNS. NO is synthesized by members of the NO synthase family, including iNOS, neuronal NOS (nNOS), and endothelial NOS (eNOS) [23]. The current study showed that MH decreased the NO secretion and iNOS expression induced by LPS stimulation. Our results suggest that MH decreases NO production by inhibiting iNOS mRNA and protein expression. In contrast, the downregulation or inhibition of COX-2 is associated with a decrease in PG production, because COX-2 is an enzyme that synthesizes PGs [18]. In this study, MH decreased the expression of COX-2 mRNA and protein. This result suggests that MH may modulate PG production.

Next, we evaluated the mechanisms underlying the anti-inflammatory effects of MH in LPS-treated BV2 cells. LPS stimulation activates Toll-like receptor (TLR) 4/MyD88, which substantially triggers the NF-κB and MAPK signaling pathways in microglia. Upon phosphorylation and degradation of IκBα, the released NF-κB translocates from the cytosol into the nucleus and induces an upsurge in the transcription of inflammatory genes [24]. In addition, among the transactivation domains of NF-κB p65 subunits, Ser536 is a well-known residue that contributes to NF-κB activity [25,26]. In our study, the LPS-activated MAPK signaling pathway was inhibited by MH treatment in microglial cells. Moreover, Western blot results showed that p-IκBα (Ser32/Ser36), p-p65 (Ser536), and nuclear p65 were detected at high levels and that IκBα was low in LPS-induced microglia, which decreased the degradation of IκBα and the translocation and phosphorylation of p65 in a dose-dependent manner. Furthermore, we confirmed the suppressive effect of MH treatment on LPS-induced NF-κB translocation using fluorescence imaging. These results suggest that MH can reduce neuroinflammatory gene expression by inhibiting the degradation of IκBα and activation of MAPK signaling, thus preventing translocation and phosphorylation of the p65 subunit.

In our study, intracellular ROS levels were lower in microglial cells treated with MH than those in the LPS control. Many previous studies have shown that ROS mediates the generation of proinflammatory molecules under stressful conditions and plays a key role in aging and the development of a variety of inflammatory diseases, such as neurodegenerative diseases. Furthermore, these molecules and their metabolites and intracellular ROS overproduction can also induce mitochondrial oxidative damage, eventually resulting in cell death [27,28].

To prevent oxidative damage during inflammation, vertebrate cells have evolved an array of antioxidant defense systems that remove ROS. Among the antioxidant genes, HO-1 is a representative antioxidant and anti-inflammatory gene that produces bilirubin and CO through heme group degradation [29,30]. The expression of HO-1 is closely associated with Nrf2, which is a key transcription factor of many cytoprotective genes, including HO-1, NAD(P)H quinone oxidoreductase (NQO)-1, and the glutamate-cysteine ligase modifier subunit (GCLM). The activity of Nrf2 is essentially involved in Keap1. Under normal conditions, Keap1 binds to Nrf2, resulting in the ubiquitination and degradation of Nrf2 [31]. Under stimulated conditions, Nrf2 liberated from cytoplasmic Keap1 is phosphorylated and subsequently transported into the nucleus, where it induces the production of HO-1 [32]. In the current study, MH treatment increased HO-1 expression. Furthermore, Nrf2 expression and Keap1 degradation were significantly induced by MH treatment for 3 h. Our results suggest that Nrf2 translocation may be accompanied by the phosphorylation of Nrf2 following MH induced-Keap1 degradation that masks the nuclear localization signal of Nrf2. Since translocated Nrf2 has transcriptional activity, it may increase the expression of HO-1, as well as of other cytoprotective genes. In contrast, we showed that the phosphorylation of CREB was induced in a dose-dependent manner by treatment with MH for 3 h. Previous studies have demonstrated that the PKA/CREB pathway is an upstream modulator as a transcription factor that binds the HO-1 promoter directly or as an inducer of Nrf2-binding proteins and activator protein-1 proteins indirectly in microglial cells [33,34]. Interestingly, a previous study showed that the abolition of CREB1 decreased PKCε-induced Nrf2 DNA binding, suggesting that CREB1 may be a transcriptional cooperator of Nrf2 [35]. Furthermore, Lee et al. reported that CREB plays a role in ROS detoxification [34]. Therefore, MH increased CREB/Nrf2/HO-1 signaling, and the activation of this signaling can reduce LPS-induced ROS production in BV2 cells. 

Menthol, a main chemical constituent extracted from *Mentha* species, has been reported to display a defense effect against inflammatory disease conditions in various tissues, including the stomach [36], lungs [37], and brain [38]. A previous study showed that menthol, administered during the initiation phase, attenuates forestomach carcinogenesis by suppressing early peripheral leukocyte blood genotoxicity, epithelium cell proliferation, and apoptosis induced by benzo(a)pyrene [36]. On the other hand, it also reported that L-menthol significantly alleviated cigarette smoke extract-induced lung injury in rats with its anti-inflammatory and antioxidative properties. L-menthol suppressed the total white blood cells and macrophages influx, as well as NF-κB and p38 MAPK pathways. The antioxidative activity of L-menthol can be indicated as a decrease of oxidative stress markers (malondialdehyde and myeloperoxidase) and encouraging the Nrf2 pathway [37]. Jian et al. reported that menthol prevented the neuroinflammatory response through MAPK, NF-κB, and AKT. Additionally, these effects of menthol led to improvement of impaired dopaminergic neurons and motor dysfunction in an LPS-induced PD rat model [38]. Based on these previous studies, the effects of menthol may be closely related to the protective effects of MH on neuroinflammatory conditions. 

Taken together, MH is a promising anti-inflammatory agent for preventing the inflammatory activation of BV2 cells through the inhibition of ROS generation and HO-1 induction. Nevertheless, our study has some limitations. Since this study evaluated the anti-inflammatory effect of MH in BV2 cells, further studies on AD drugs are required to investigate the inhibitory effect of MH on neurodegeneration in other cell lines. Furthermore, it is necessary to evaluate its anti neuroinflammatory effects in vivo. However, it should be further explored to elucidate the active compounds in MH and to carry out more specific mechanistic studies with the compounds to consolidate evidence that MH could regulate neuroinflammation by increasing the antioxidative pathway. 

## 5. Conclusions

In this study, the inflammatory response of MH and possible molecular mechanisms in microglia stimulated with LPS were evaluated. The results showed that MH inhibited the production of proinflammatory enzymes and mediators. Moreover, MH inhibited the production of ROS and phosphorylation of MAPK and NF-κB. Additionally, MH activated HO-1 and its transcriptional factors, the CREB and Nrf2 signaling pathways. Our results suggest that MH could be used as a potential therapeutic agent for neuroinflammatory diseases due to the attenuation of ROS and inflammation signaling related to MAPK and NF-κB through the induction of CREB/Nrf2/HO-1-mediated antioxidant signaling in LPS-stimulated BV2 microglial cells. 

## Figures and Tables

**Table 1:** Primers used for RT-qPCR.

Target Gene	Primer Sequence (5′-3′)	Gene Bank Accession Number
COX-2	F: TGAGTACCGCAAACGCTTCTC R: TGGACGAGGTTTTTCCACCAG	NM_011198.4
HO-1	F: TGAAGGAGGCCACCAAGGAGG R: AGAGGTCACCCAGGTAGCGGG	NM_010442.2
IL-1β	F: ATGGCAACTGTTCCTGAACTCAACT R: CAGGACAGGTATAGATTCTTTCCTTT	NM_008361.4
IL-6	F: TCCAGTTGCCTTCTTGGGAC R: GTGTAATTAAGCCTCCGACTTG	NM_001314054.1
iNOS	F: GGCAGCCTGTGAGACCTTTG R: GCATTGGAAGTGAAGCGTTTC	NM_001313922.1
MCP1	F: ACCTGGATCGGAACCAAATG R: CCTTAGGGCAGATGCAGTTT	NM_011333.3
TNF-α	F: TTCTGTCTACTGAACTTCGGGGTGATCGGTCC R: GTATGAGATAGCAAATCGGCTGACGGTGTGGG	NM_001278601.1
36B4	F: ACCTCCTTCTTCCAGGCTTT R: CTCCAGTCTTTATCAGCTGC	NM_007475.5

F—forward and R—reverse.

## Data Availability

All the data are available within the article.
